# SOX17 is a tumor suppressor in endometrial cancer

**DOI:** 10.18632/oncotarget.12582

**Published:** 2016-10-12

**Authors:** Yongli Zhang, Wei Bao, Kai Wang, Wen Lu, Huihui Wang, Huan Tong, Xiaoping Wan

**Affiliations:** ^1^ Department of Obstetrics and Gynecology, Shanghai First Maternity and Infant Hospital, Tongji University School of Medicine, Shanghai, China; ^2^ Department of Obstetrics and Gynecology, International Peace Maternity & Child Health Hospital Affiliated to Shanghai Jiao Tong University School of Medicine, Shanghai, China; ^3^ Department of Obstetrics and Gynecology, Shanghai First People's Hospital Affiliated to Shanghai Jiao Tong University, Shanghai, China

**Keywords:** SOX17, MAML3, endometrial cancer, β-catenin, Wnt signaling

## Abstract

β-catenin is a key regulatory factor for the Wnt signaling pathway. SOX17 is an important β-catenin inhibitor, while MAML3 is a co-activator of β-catenin-mediated transcription. Out of 120 endometrial cancer (EC) patients, we found that those with tumors expressing higher SOX17 (n=68) had longer recurrence-free survival (*P*=0.024), while higher MAML3 expression (n=76) was associated with shorter recurrence-free survival (*P*=0.022). Immunohistochemical and immunoprecipitation analyses revealed that SOX17 and MAML3 co-localized in EC cell nuclei, and the MAML3 C-terminal region was necessary for SOX17 binding. SOX17 regulated *MAML3* transcription via binding to the *MAML3* promoter, decreasing Wnt pathway protein expression and suppressing EC cell growth and colony formation *in vitro*. In nude mice, SOX17 over-expression inhibited tumor growth, and co-inhibition or co-overexpression of SOX17 and MAML3 rescued this response. Our results suggest that decreasing SOX17 levels may promote EC development and progression, and that by downregulating MAML3 expression and Wnt signaling, SOX17 acts as a tumor suppressor that may improve outcome in patients with EC.

## INTRODUCTION

Endometrial cancer (EC) is the most common gynecologic malignancy, with an estimated 47,130 new cases and 8,010 deaths occurring in 2014 [1]. Two clinicopathological variants are distinguished: estrogen-dependent type I and estrogen-independent type II, with type I showing abnormal β-catenin activity and nuclear accumulation [3], and type II exhibiting TP53 mutations [2]. Aberrant β-catenin activation can lead to constitutive transcriptional activation of β-catenin/TCF target genes, enhancing cellular proliferation [4, 5].

SOX17 (SRY-box containing gene 17) is a member of the SRY-related High Mobility Group (HMG) box transcription factor superfamily and regulates a variety of developmental processes and diseases [6]. SOX17 encodes a 414-amino-acid (aa) protein with an HMG box, which was implicated in embryogenesis [7], and likely plays an important role in carcinogenesis [8]. SOX17 expression is reduced in some cancer cells [9–11] and was correlated with poor prognoses [11–13]. SOX17 negatively regulates β-catenin/TCF transcription activity in Wnt/β-catenin signaling [9, 14, 15], and can bind the promoter region of *Maml3* [17]. While it is reportedly mutated in 8% of ECs [16], the role of SOX17 in this cancer is still unclear.

MAML3 is a critical transcriptional co-activator in the Notch signaling pathway, and is encoded by a family of Mastermind like (MAML) genes [18]. MAML3 is a co-activator for β-catenin mediated transcription and dramatically increased β-catenin transcriptional activity on promoters containing TCF-binding sites. It appears that MAML3 is recruited by β-catenin to Wnt target gene promoters (for example, Cyclin D1 and c-Myc) independently of Notch signaling [19].

Based on these observations, we hypothesized that SOX17 downregulates MAML3, modulating nuclear β-catenin and antagonizing Wnt signaling in EC. We assessed the functional significance of SOX17 in EC *in vivo* and *in vitro* via MAML3 and SOX17 overexpression and downregulation.

## RESULTS

### SOX17 expression is correlated with MAML3 expression

**Figure 1:**
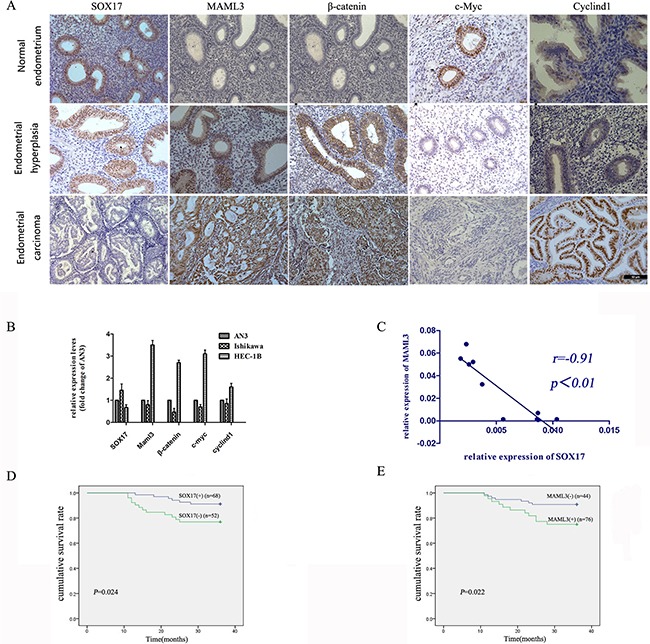
SOX17 expression is negatively correlated with MAML3 in EC patient samples and cell lines IHC and real-time PCR were used to detect SOX17 and MAML3, respectively, in different samples. IHC analysis of SOX17, MAML3 and Wnt pathway proteins in normal endometrium, atypical hyperplasia and endometrial cancer tissues **A.** Error bar=50um, *P<0.05. Real-time PCR analysis of SOX17, MAML3 and Wnt pathway proteins in endometrial cancer cells **B.** Low SOX17 levels were correlated with increased MAML3, CyclinD1, c-Myc and β-catenin expression in EC cell lines. SOX17 and MAML3 were inversely correlated in human endometrial cancer cells (R=−0.91, *P*=0.001) **C.** In Kaplan-Meier analyses, patients with low SOX17-expressing (n=52, *P*=0.024) **D.** or high MAML3-expressing (n=76, *P*=0.022) **E.** tumors had poor overall survival. Results represent at least three separate experiments.

Immunohistochemical (IHC) analyses of human normal endometrium (n=30), atypical hyperplasia (n=30) and EC tumor specimens (n=60) showed that SOX17 expression was very low or absent in late EC stages, with high expression of Wnt pathway proteins. MAML3 expression was high in well-, moderately- and poorly- differentiated EC tissue samples (Figure 1A). SOX17 and MAML3 were negatively correlated in all tissues (R = −0.340, *P*=0.001). These observations suggested that decreased SOX17 expression and subsequent high MAML3 expression, along with associated Wnt signaling abnormalities, could promote EC development and progression.

Real-time PCR results showed reduced SOX17 expression in HEC-1B cells as compared with AN3CA cells with higher MAML3 levels; this was reversed in Ishikawa cells (Figure 1B). SOX17 and MAML3 levels were negatively correlated in EC cells (R = −0.91, *P*=0.001) (Figure 1C).

The Kaplan-Meier analysis was used to assay recurrence-free survival differences associated with high or low SOX17 and MAML3 expression. Out of 120 EC patients, those with tumors expressing higher SOX17 (n=68) had significantly longer recurrence-free survival (*P*=0.024) (Figure 1D), while higher MAML3 expression (n=76) was associated with shorter recurrence-free survival (*P*=0.022) (Figure 1E).

Together, these results suggested a functional interaction between SOX17 and MAML3 during EC progression.

### SOX17 directly binds MAML3

**Figure 2:**
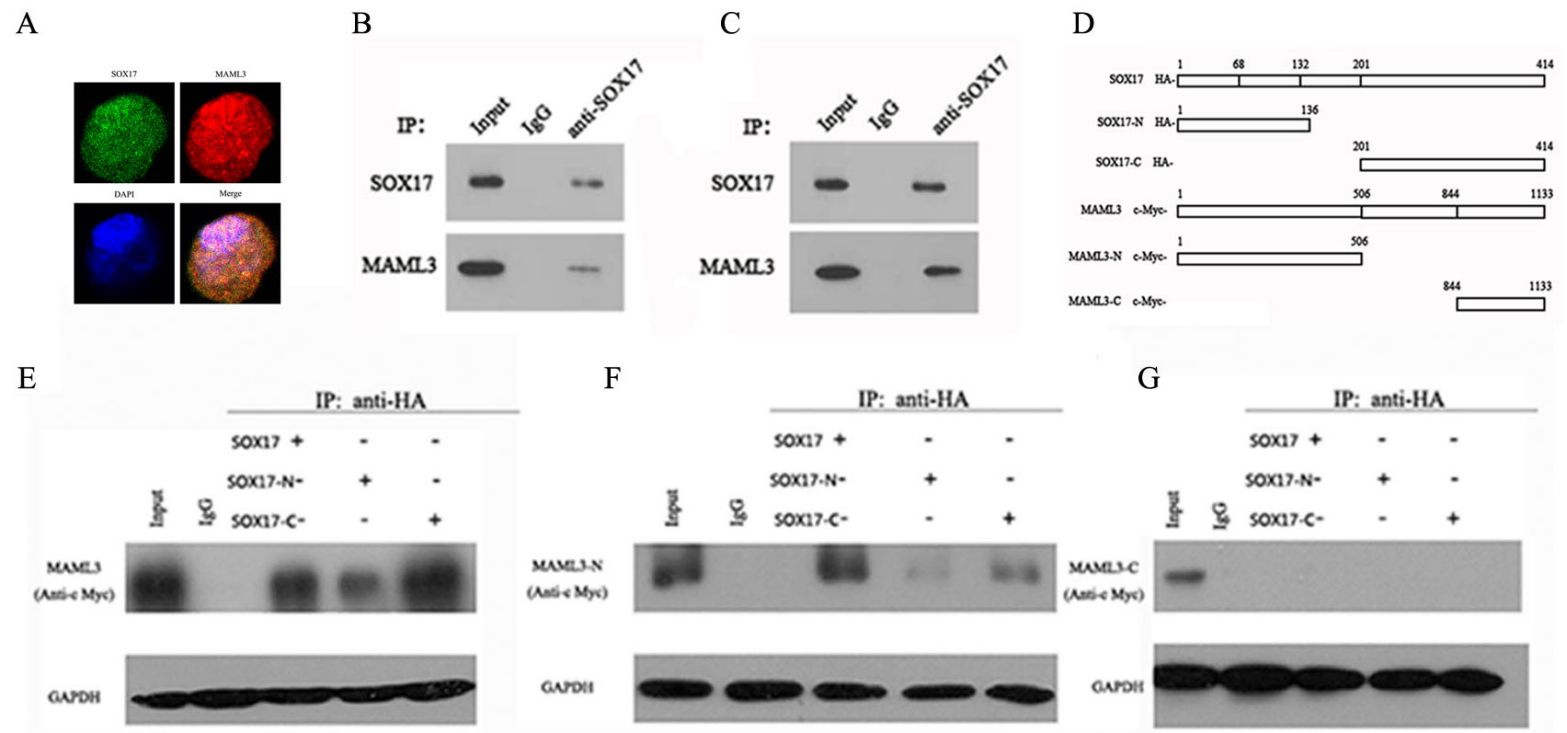
SOX17 directly interacts with MAML3 HEC-1B cells overexpressing SOX17 were seeded on 96-well plates. IF analysis showed that MAML3 (red) co-localized with SOX17 (green) in HEC-1B cell nuclei **A.** HEK-293 cells were transfected with SOX17 and MAML3 cDNA. Lysates were prepared and SOX17 antibody was used for Co-IP assays, followed by western blot (WB) analysis using MAML3 antibodies **B.** HEC-1B cells were transfected with SOX17 cDNA and analyzed via Co-IP and WB **C.** Different fragments of SOX17 and MAML3 **D.** were expressed in HEK-293T cells and evaluated by WB using HA and c-Myc antibodies.

SOX17 and MAML3 nuclear co-localization was confirmed using a laser scanning confocal microscope (Figure 2A), suggesting that SOX17 might target MAML3. Whole cell extracts, HEK293 cells transfected with vector expressing SOX17/MAML3 and HEC-1B cells, were prepared. Co-immunoprecipitation (Co-IP) analyses showed that exogenous and endogenous SOX17 associated with MAML3 (Figure 2B & 2C).

We then developed plasmids encoding either the N- or C-termini of SOX17 or MAML3, with HA or c-Myc tags (Figure 2D). HEK293 cells were cotransfected with SOX17 and MAML3 vectors and immunostained with antibodies against the HA- or c-Myc tags (Figure 2E, 2F & 2G). Co-IP assay showed that the SOX17 full-length protein, N-terminus (aa 1-132) and C-terminus (aa 201-414) could each bind the full-length MAML3 protein and N-terminus (aa 1-506), but not the MAML3 C-terminus (aa 844-1134). This suggested that the 844-1134 aa region was important for binding of MAML3 and SOX17.

### SOX7 inhibits MAML3 transcription

**Figure 3:**
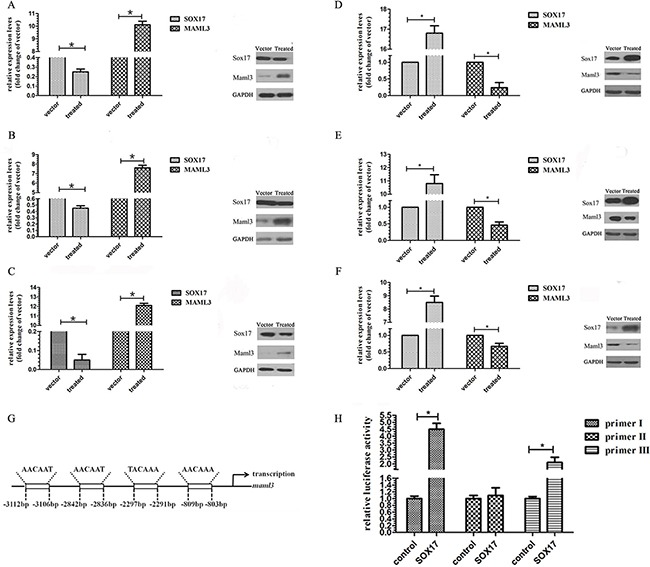
SOX7 inhibits MAML3 transcription Real-time PCR and western blot assays were used to characterize MAML3 expression in SOX17-silenced AN3CA **A.** HEC-1B **B.** and Ishikawa **C.** cells and in SOX17-overexpressing AN3CA **D.** HEC-1B **E.** and Ishikawa **F.** cells. SOX17 putative binding site located −803 to −809 bp from the transcription start site in the *MAML3* promoter region. **G.** ChIP analysis of SOX17 binding to *MAML3* in HEC-1B cells **H.**

Western blot and real-time PCR results showed that decreased SOX17 expression increased MAML3 mRNA and protein levels in AN3CA (Figure 3A), Ishikawa (Figure 3B), and HEC-1B cells (Figure 3C). Similarly, increased SOX17 expression decreased MAML3 mRNA and protein levels (Figure 3D, 3E & 3F).

Multi-genome Analysis of Positions and Patterns of Elements of Regulation (MAPPER, http://snpper.chip.org/mapper/mapper-main) identified a putative SOX17 binding site located −803 to −809 bp from the transcription start site in the *MAML*3 promoter region (Figure 3G). A chromatin immunoprecipitation (ChIP) assay showed that SOX17 could occupy the *MAML*3 promoter (Figure 3H). DNA immunoprecipitated via an anti-SOX17 antibody was amplified using the *MAML*3 promoter primers. The promoter fragment was specifically co-immunoprecipitated using the anti-SOX17 antibody, but not the control IgG, and increased SOX17 specifical binding was observed in SOX17-transfected HEC-1B cells.

### SOX17 controls MAML3-induced β-catenin transcription activity downregulation

We evaluated SOX17, MAML3, β-catenin, CyclinD1 and c-Myc expression by IHC analysis in paraffin-embedded human ECsamples (Figure 1A). SOX17 expression was not detected in 24 of60 EC cases. Of these 24 cases, 22 were nuclear β-catenin positive. Thus, nuclear accumulation of β-catenin is associated withnegative SOX17 in endometrial cancer (R = −0.392, *P*=0.001), suggesting that SOX17 could influence Wnt/β-catenin signaling.

**Figure 4:**
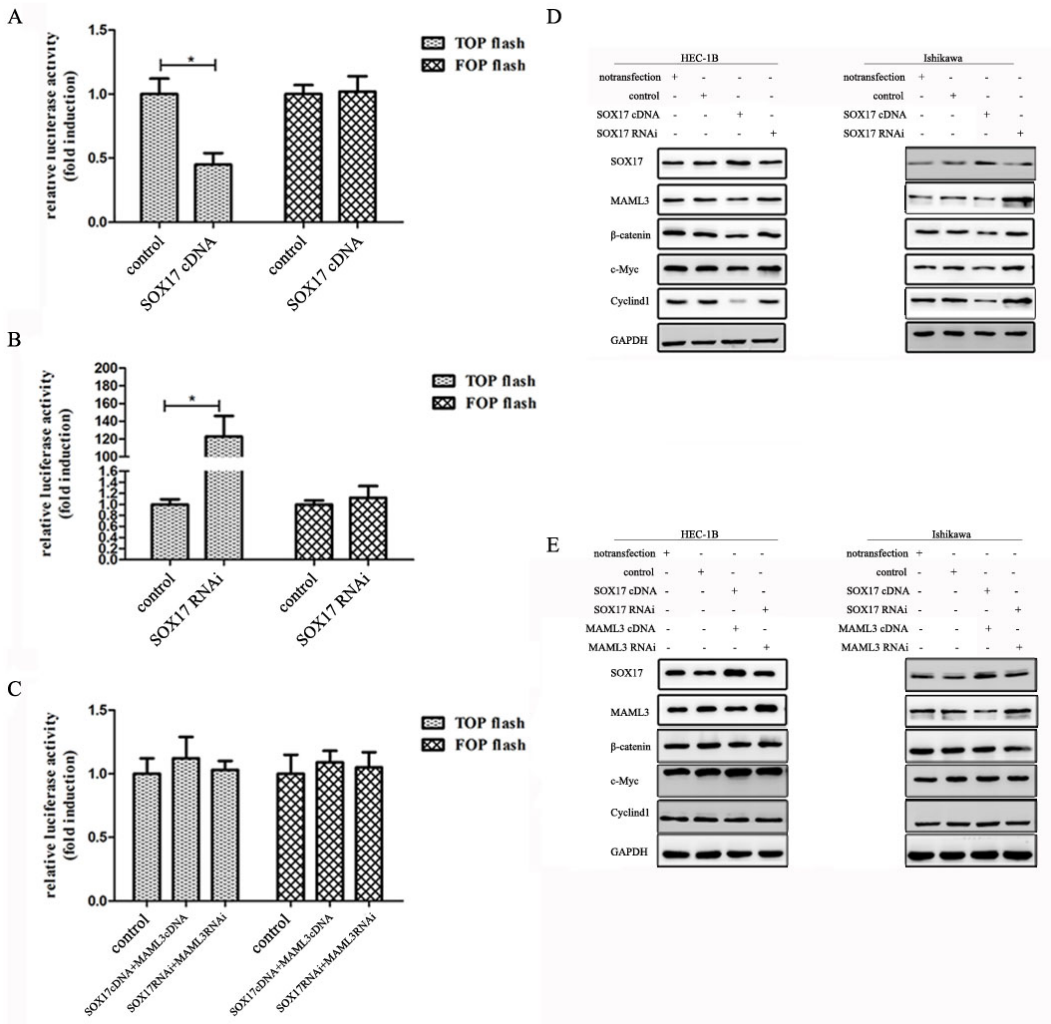
SOX17 antagonizes the canonical Wnt pathway in HEC-1B cells Cells were transfected with or without SOX17 cDNA together with the TCF-luciferase reporter (TOPflash) or mutant TCF-luciferase reporter (FOPflash) for 24 h. SOX17 knockdown repressed TOPflash activation in HEC-1B cells **A.** SOX17 overexpression increased TOPflash activation in HEC-1B cells **B.** SOX17/MAML3 co-transfection did not affect basal TOPflash activity **C.** HEC-1B and Ishikawa cells were transfected with SOX17 cDNA or shRNA alone or together with the MAML3 plasmid **D.** & **E.** Immunoblotting was performed on 10 μg of whole cell lysates. GAPDH was used as loading control.

The β-catenin promoter luciferase reporter, TOPflash, was co-transfected into HEK293 cells with SOX17cDNA or SOX17 shRNA. TOPflash activity was decreased by co-transfection of SOX17 cDNA and increased by SOX17 shRNA. We then introduced point mutations in TOPflash (now called FOPflash) using site-directed mutagenesis by base substitution. The mutants were no longer responsive to SOX17 (Figure 4A & 4B). FOPflash was also unresponsive to SOX17 when co-transfected with both SOX17 cDNA and MAML3 cDNA or SOX17 shRNA and MAML3 shRNA (Figure 4C). Together, these results indicated that SOX17 suppresses β-catenin/TCF transcription activity by down-regulating MAML3.

Further validating this observed SOX17 inhibitory effect, MAML3 expression was down-regulated by SOX17 as measured by western blot. c-Myc and CyclinD1 were down-regulated by SOX17 indirectly (Figure 4D). In contrast, when we increased or knocked down SOX17 and MAML3 together, β-catenin and downstream CyclinD1 and c-Myc levels were not different from control groups (Figure 4E).

### SOX17 overexpression inhibits EC cell proliferation *in vitro*

**Figure 5:**
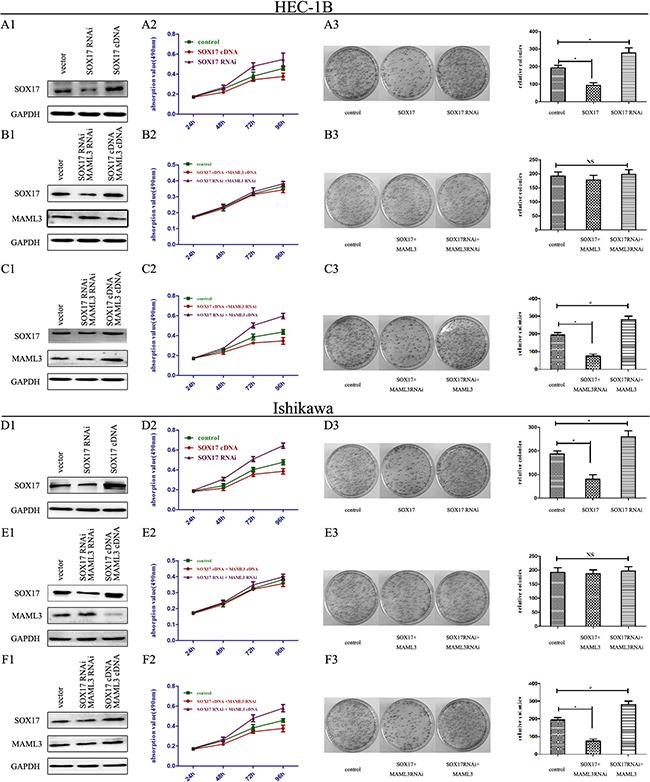
SOX17 suppressed EC cell proliferation *in vitro* HEC-1B cells were transfected with either SOX17 cDNA or shRNA plasmids. SOX17 expression was analyzed by western blot **A1.** Cell viability was assessed by MTT assay daily for 4 days **A2.** Colony formation assays were performed on HEC-1B cells for 2 weeks. (*P<0.05) **A3.** HEC-1B cells were co-transfected with SOX17/MAML3 shRNA or cDNA plasmids, and analyzed by western blot **B1.** Cell viability was assessed by MTT assay daily for 4 days **B2.** Colony formation assays were performed for 2 weeks (*P<0.05) **B3.** HEC-1B cells were transfected with SOX17 cDNA/MAML3 shRNA or SOX17 shRNA/MAML3 cDNA plasmids and analyzed via western bolt **C1.** Cell viability was assessed by MTT assay daily for 4 days **C2.** Colony formation assays were performed for 2 weeks (*P<0.05) **C3.** Ishikawa cells were analyzed following the same treatments (*P<0.05) **D1.-F3.**

We transfected SOX17 cDNA or SOX17 shRNA into HEC-1B and Ishikawa cells (Figure 5A1 & 5D1). MTT analysis showed that SOX17 cDNA-transfectedcells grew much slower than controls (Figure 5A2 & 5D2). Colony formation assays also revealed that SOX17 cDNA-transfected cells formed smaller and fewer colonies than controls (Figure 5A3 & 5D3). Conversely, SOX17 shRNA-transfectedcells had higher proliferation rates and formed larger and more colonies than controls.

Next, because SOX17 overexpression reduced MAML3 levels, we then transfected MAML3 cDNA into SOX17 over-expressing HEC-1B and Ishikawa cells to rescue MAML3 expression (Figure 5B1 & 5E1). MTT analysis showed that SOX17/MAML3-overexpressing cells grew at the same rate as controls, and colony formation was not different from controls (Figure 5B2, 5B3, 5E2 & 5E3). When we knocked down SOX17 and MAML3 together, we observed the same results. Together, these results indicated that SOX17 overexpression inhibited cell proliferation by downregulating MAML3.

Finally, HEC-1B and Ishikawa cells co-transfected with MAML3 shRNA and SOX17 cDNA grew slower the controls and formed smaller colonies (Figure 5C1-5C3 & 5F1-5F3).

### SOX17 overexpression inhibits EC cell proliferation *in vivo*

**Figure 6:**
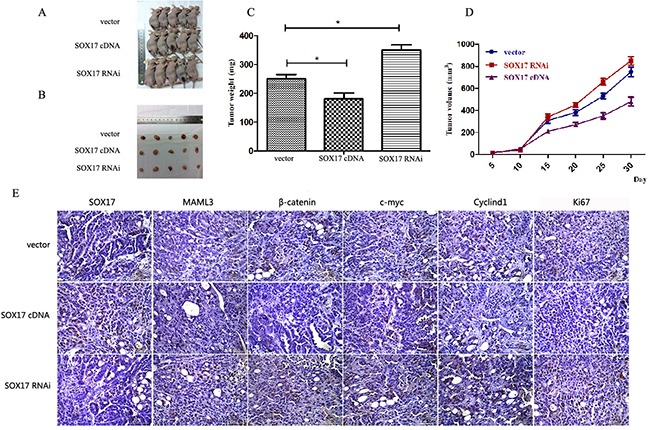
SOX17 suppressed tumor growth *in vivo* Tumors from xenograft-transplanted nude mice 32 days after subcutaneous injection of SOX17-overexpressing, silenced or control HEC-1B cells **A.** Weights of xenografts 32 days after cell injection (*P<0.05) **B.** *In vivo* tumor growth curves for HEC-1B cells **C.** Paraffin-embedded sections obtained from xenografts **D.** IHC staining shows increased MAML3, β-catenin, CyclinD1, c-Myc and Ki67 expression in the SOX17 knockdown group compared to controls.

**Figure 7:**
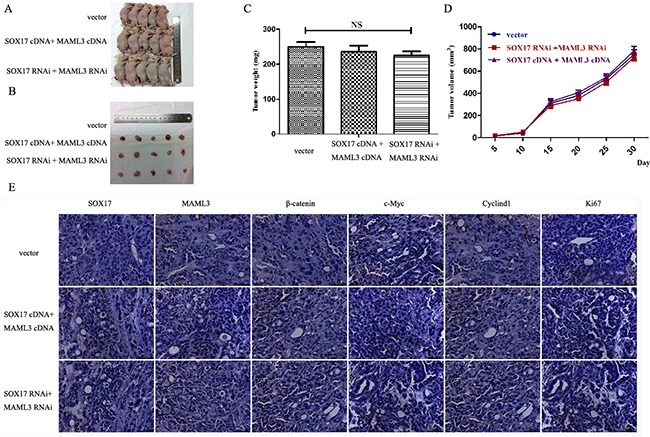
SOX17 suppressed tumor growth by downregulating MAML3 *in vivo* Tumors from xenograft-transplanted nude mice 32 days after subcutaneous injection of SOX17 or MAML3-overexpressing, silenced or control HEC-1B cells **A.** Weights of xenografts 32 days after cell injection (*P<0.05) **B.** *In vivo* tumor growth curves for HEC-1B cells **C.** Paraffin-embedded sections obtained from xenografts **D.** IHC staining shows that MAML3, β-catenin, CyclinD1, c-Myc and Ki67 levels were not significantly different compared to controls.

Stably transfected cells were subcutaneously transplanted into BALB/c nude mice. Cells were suspended at 5×10^6^ cells/ml and 100 μl was injected into the flanks of nude mice (n=5). We measured tumor sizes starting 5 days post-injection using the formula (*W^2^* × *L*) every 5 days for 32 days. Mice were then euthanized and tumors were excised. Tumor weights (Figure 6C) and sizes (Figure 6D) from SOX17-overexpressing cells were reduced compared to controls. Tumors from the SOX17-overexpression group exhibited significantly lower β-catenin, CyclinD1 and c-Myc levels compared with controls, as measured by western blot (Figure 6E). When SOX17 and MAML3 were co-overexpression or co-downregulated transfected tumors grew similarly to controls (Figure 7) (*P*>0.05)

## DISCUSSION

In this study, we evaluated SOX17 expression in EC patient tumor samples and cell lines. High SOX17 levels were observed in benign endometrial tumor tissues, with low expression in malignant EC tissues. As a canonical Wnt antagonist, SOX17 is epigenetically inactivated by promoter methylation in many cancers [10, 12, 14], regulating proliferation, the cell cycle and angiogenesis during cancer progression [20–22]. Our results indicated that SOX17 was an important tumor suppressor in EC. SOX17 overexpression in EC cell lines confirmed that the cellular and molecular alterations observed in EC tumors were indeed due to SOX17 levels. Thus, accumulation of β-catenin in the nucleus is associated with low-to-noSOX17 expression in endometrial cancer.

SOX17 inhibits β-catenin/TCF-dependent transcription [23] and inhibits cancer cell proliferation [15] and colony formation [21]. In our study, IHC analyses revealed inhibition of β-catenin/TCF transcription activity in SOX17-overexpressing tumors. Furthermore, *in vivo* studies demonstrated significantly reduced growth in tumors overexpressing SOX17 compared with controls.

Tight control of nuclear β-catenin levels is key for regulation of Wnt/β-catenin signaling, and Wnt/β-catenin pathway dysregulation plays an essential role in type I EC in particular [24]. One consequence of aberrant Wnt signaling is constitutive transcriptional activation of β-catenin/TCF target genes associated with tumorigenic processes, such as sustained cellular proliferation in the absence of growth signals [23].

c-Myc is reportedly an important effector of β-catenin, while Cyclin D1 is a critical β-catenin target [25]. Our observations revealed that SOX17 overexpression correlated with reduced CyclinD1 and c-Myc levels, suggesting that SOX17 suppresses tumor growth through inhibition of β-catenin/TCF activity and the Wnt signaling pathway.

Our investigations revealed that SOX17 was inversely correlated with MAML3 expression in EC cell lines and tumor tissues. Several findings supported direct binding of SOX17 to the *MAML3* promoter as the mechanism for inhibiting β-catenin/TCF activity and Wnt signaling. First, SOX17 decreased *MAML3* mRNA and protein levels in EC cell lines (Figure 3A-3F). This suggests that MAML3 expression is predominately regulated by SOX17 at the transcription level. Second, ChIP analysis demonstrated that two regions in the endogenous *MAML3* promoter bound SOX17 (Figure 3H). The present study provides evidence that suppression of MAML3 transcription by SOX17, via binding the *MAML3* promoter, may reduce cell proliferation, leading to favorable outcomes in EC patients.

Emerging evidence implicates MAML proteins as key transcriptional co-activators in Wnt signal transduction pathways, including colon carcinoma survival [19]. MAML3 participated in Wnt signaling by modulating β-catenin/TCF activity and interacted with β-catenin *in vitro* and *in vivo*, dramatically increasing β-catenin transcriptional activity on promoters containing TCF-binding sites [26]. SOX17 can bind the *MAML3* promoter and MAML3 rescues the effect of SOX17 on β-catenin and target proteins in Wnt pathway. This suggests that SOX17 silences MAML3 expression and regulates expression of its critical target genes involved in EC pathogenesis.

The current study investigated the functional role of SOX17 in EC pathogenesis. Our observations suggest that decreased SOX17 levels may promote EC development and progression, and that SOX17 is a tumor suppressor in EC. We provide evidence that SOX17 downregulates MAML3 and Wnt signaling, which may reduce tumor cell proliferation and improve patient outcome.

## MATERIALS AND METHODS

### Patients

The protocols used in the study were approved by the Hospital's Protection of Human Subjects Committee. Samples were acquired with written informed consent from the Shanghai First Maternity and Infant Hospital affiliated with the Tongji University School of Medicine.

Paraffin-embedded tissues from 120 patients were included in the study between December 2008 and December 2010, including human normal endometrium (n=30), atypical hyperplasia (n=30) and EC tumors (n=60). The average age of patients with EC was 51.8±2.8 years (mean±SD; median, 53 years; range, 42–61 years). Tumor stages (I–III) and histological grades (G1–G3) were established according to the criteria of the International Federation of Gynecology and Obstetrics (FIGO) surgical staging system (2009) [27]. None of the patients had undergone hormone therapy or radiotherapy before surgery.

### Cell lines and transfections

The human EC cell lines Ishikawa, HEC-1B and AN3CA, were obtained from and maintained as recommended by the China Center for Type Culture Collection (CCTCC). Cells were cultured in Dulbecco's modified Eagle's medium (DMEM) with 10% fetal bovine serum (FBS, Wisent, Quebec, Canada) at 37°C with 5% CO2. Transfections were performed with Lipofctamine 2000 transfection reagent (Invitrogen, Carlsbad, USA) in 6-well plates following the manufacturers' protocols. For transfection efficiency analysis, prior to transfection, 1×10^6^ cells per well were seeded and then harvested at 24 h for real-time PCR analysis, or at 72 h for western blot analysis. SOX17-overexpressing and knockdown cell lines were established as previously described [28]. For immunofluorescence staining, a low density of transfected cells (about 1×10^5^) was seeded and analyzed at 24 h post transfection. All experiments were repeated three times unless stated otherwise.

### Immunohistochemistry

IHC staining was performed as previously described [29]. Antibodies included: SOX17 (abcam, Cambridge, UK), MAML3 (abcam), β-catenin (abcam), cyclinD1 (abcam), c-Myc (abcam) and Ki67 (abcam). Scoring was performed by two independent pathologists blinded to clinicopathologic data. Protein staining was evaluated as described [30].

### RNA extraction and analysis

Total RNA extraction and reverse transcription (RT) reactions were performed as described previously [31]. Real-time PCR was performed using a standard protocol from the SYBR Green PCR kit (Toyobo, Osaka, Japan). GAPDH was used as the reference. Primer pairs used were as follows: SOX17 forward: ‘5- ATCCTCAGACTCCTGGGTTT-3′, reverse: ‘5-ACTGTTCAAGTGGCAGACAAA-3′; MAML3 forward: ‘5-TGAAGAGAAGAAGGA-3′, reverse: ‘5-GTG CGAAGGGAGAGTAGAAG-3′; GAPDH forward: ‘5-GGCTCCCTTGGTATATGGT-3′, reverse: ‘5-TTGATT TTGGAGGGATCTCG-3′.

### Immunoprecipitation

Immunoprecipitation was performed as previously described [32]. Protein A+G Agarose beads (40μl per reaction) were added (Beyotime, Jiangsu, China). Immunoprecipitated proteins were then subjected to SDS-PAGE and western blot analysis. Antibodies used for IP were as follows: SOX17 (abcam), MAML3 (abcam), HA (abcam) and c-Myc (abcam).

### Immunoblotting

Western blot analysis to assess protein expression was performed as previously described [33]. Protein concentrations were determined using the BCA protein assay kit (Bioteke, Beijing, China). Primary antibodies included: GAPDH (Sigma, ST Louis MO, USA); all others were described previously.

### Chromatin immunoprecipitation assay

ChIP assay was performed using a Chromatin Immunoprecipitation (ChIP) Assay Kit (Millpore, Massachusetts, USA) as described previously [34]. DNA was fragmented by sonication (JY02-II Ultrasonic cell lyser, Ningbo, China) to about 400 to 600 bp. PCR and RT-qPCR were performed using MAML3 primer I: forward: 5′- GTTTTGGTCATGACAAACTGTG-3′, reverse: 5′- CACTGTACATAATGATTGTTGTTAC-3′; Primer II: forward: 5′- GTTTTGGTCATGACAAACTGTG-3′, reverse: 5′- GGTGCTAATGACAGCAGTAATTAAG -3′; and Primer III: forward: 5′- TGTTAGTCTCACCTCCAAGTCC -3′, reverse: 5′- CACTGTACATAATGATTGTTGTTAC -3′. ChIP analysis was performed 24 h after transfection.

### Plasmids

The SOX17cDNA plasmid was purchased from Origene (Maryland, USA) and the MAML3cDNA plasmid was purchased from Genecopoeia (California, USA). RNAi was performed as previously described [35]. siRNA targeting SOX17 and MAML3 were as follows: SOX17siRNA: ‘5- GGTATATTACTGCAACTAT-3′; and MAML3siRNA: ‘5-CAGGATATAGCAGCCGTAA-3′. shRANs were cloned into the pCMV6-Entry vector which was purchased from Origene (USA). Positive clones were identified by PCR and sequenced.

### Cell proliferation and clonogenicity assays

Cells were plated for 24 h and transfected with SOX17 plasmid or MAML3 plasmid. The number of viable cells was determined at 24, 48, 72 and 96 h using the 3-(4,5-dimethylthiazol-2yl)-2,5-diphenyltetrazolium bromide (MTT) assay as described [31]. For the anchorage-dependent colony-forming assay, cells (4×10^2^) were transfected and incubated for 14 days, fixed with 100% methanol and stained with hematoxylin. Colonies (>50 cells) were counted manually and plotted as described [36].

### Luciferase assays

Cells were seeded at 1×10^5^ cells/well in a 24-well cell plate one day prior to transfection with Superfect according to the manufacturer's protocol (Tiangen Biotech co LTD, Beijing, China). Luciferase activity was normalized for transfection efficiency using the mutant promoter reporter plasmid, FOPflash (vs. TOPflash, the un-mutated plasmid), as an internal control [37].

### Nude mice

5×10^6^ HEC-1B cells stably overexpression SOX17 or with SOX17 knocked down were implanted subcutaneously into 4–6-week-old BALB/c nude mice purchased from the Shanghai Laboratory Animal Center (SLAC) (Shanghai, China). Tumor growth was measured using a digital caliper every 5 days for 32 days. Tumor weight was measured when mice were sacrificed on day 32.

### Statistical analysis

All experiments were repeated in triplicate. Data were expressed as the mean±SD. Statistical significance between two groups was determined by Student's *t*-test. Associations between SOX17 expression and clinicopathological parameters were examined by the Chi-square test. *P*<0.05 was considered statistically significant.
